# COVID-19 as a trigger of cerebral venous sinus thrombosis in a patient with autoimmune hyperthyroidism: a case report

**DOI:** 10.1186/s41983-022-00476-8

**Published:** 2022-04-11

**Authors:** Rocksy Fransisca V. Situmeang, Reza Stevano, Ratna Sutanto

**Affiliations:** 1 Department of Neurology, Siloam Hospitals Lippo Village, Tangerang, Banten Indonesia; 2 Faculty of Medicine, Pelita Harapan University, Jl. Boulevard Jend. Sudirman No. 20, Lippo Karawaci, Tangerang, Banten 15810 Indonesia; 3 Department of Radiology, Siloam Hospitals Lippo Village, Tangerang, Banten Indonesia

**Keywords:** Cerebral venous sinus thrombosis, COVID-19, Hyperthyroidism, Stroke, Case report

## Abstract

**Background:**

Cerebral venous sinus thrombosis (CVST) composes an uncommon subtype of stroke caused by thrombotic occlusion of the cerebral venous system and tends to occur in hypercoagulable states. Albeit exceedingly rare, autoimmune hyperthyroidism and COVID-19 has been implicated as rare risk factors for CVST. As both conditions are capable of inducing degrees of inflammation and hypercoagulability, we postulate that COVID-19 could trigger CVST by superimposing endotheliitis and inflammation on the hypercoagulable and hypofibrinolytic state of hyperthyroidism.

**Case presentation:**

We report the case of an adult male with headache, fever, nausea, vomiting, and previously known autoimmune hyperthyroidism. Diagnostics revealed elevated inflammatory and hypercoagulability markers, free T4, low TSHs, and positive SARS-CoV-2 PCR. Neuroimaging demonstrated an acute intracerebral and subdural hemorrhage attributable to cerebral sinus thromboses. A diagnosis of CVST with associated COVID-19 and autoimmune hyperthyroidism was established, and anticoagulation therapy was initiated. Follow-up examination revealed complete symptomatic resolution and regression of thrombosis.

**Conclusions:**

Clinicians should be aware that even mild COVID-19 could precipitate CVST, especially in presence of other risk factors. Further studies should be conducted to evaluate the effects of mild COVID-19 on existing prothrombic states, including autoimmune hyperthyroidism. Furthermore, a high index of suspicion towards a secondary cause must be maintained for headaches in COVID-19, as it may indicate a serious etiology, including CVST.

## Introduction

Cerebral venous sinus thrombosis (CVST) is an uncommon subtype of stroke caused by thrombotic occlusion of the cerebral veins, constituting 0.5–1% of strokes. CVST occurs in hypercoagulable states, mostly due to multiple factors, such as inborn prothrombic conditions, oral contraceptive use, infection, and systemic disease [1, 2]. Hyperthyroidism has been implicated as a rare risk factor for CVST, wherein previous studies found it present in 1.9–7.1% of CVST patients [3, 4]. In COVID-19, CVST is extremely rare, estimated affecting 4.5 per 100,000 cases [5]. We report a case of CVST in an adult male with autoimmune hyperthyroidism and mild COVID-19, wherein CVST was likely caused by the combination of both entities. Furthermore, we highlight that a high index of suspicion must be maintained in evaluating a COVID-19 patient with headache, as it may herald a secondary cause.

## Case

A 37-year-old Southeast Asian male presents with a 4-day history of remittent fever and headache, with abrupt worsening 1-day following admission. The headache was predominantly right sided, described as stabbing, of moderate intensity (VAS 5/10), and was associated with nausea and vomiting. Previous medical history was significant for autoimmune hyperthyroidism, for which the patient routinely took oral thiamazole. The patient had not undergone COVID-19 vaccination. Upon physical examination, the patient was febrile and tachycardic, but other findings were within normal limits. The neurological examination was non-significant. The patient initially tested negative with a SARS-CoV-2 antigen assay and was admitted to inpatient care and given analgesics (paracetamol, ketorolac). One day into care, there was an abrupt worsening of headache (VAS 8/10).

**Fig. 1:**
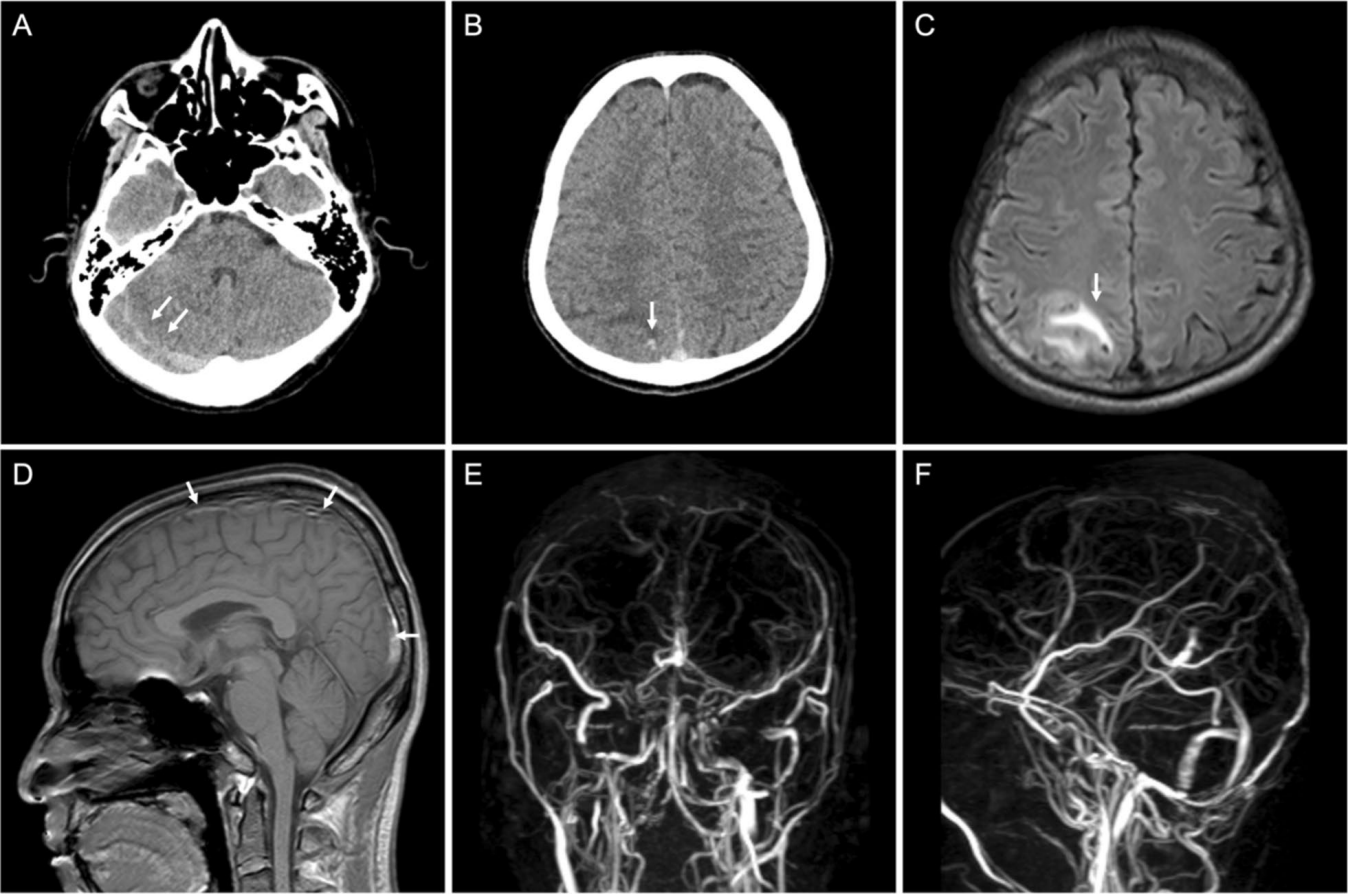
Non-contrast head CT demonstrating SDH of the posterior fossa with an approximate diameter of 0.6 cm (**A**), and small parasagittal ICH of the right parietal lobe surrounded by perifocal edema (**B**). Head MRI: FLAIR sequence reveals perifocal edema surrounding the site of hemorrhage (**C**), and T1-weighted MRI reveals thrombosis of the superior sagittal sinus (**D**). Head MRV (phase-contrast technique) demonstrates thrombosis of the superior sagittal sinus, right transverse sinus, right sigmoid sinus, and right sinus of trolard (**E**, **F**)

Diagnostics were significant for elevated CRP (19 mg/L), increased free T4 (> 7.77 ng/dL) with low TSHs (0.01 μIU/mL), reduced aPTT (23.20 s), increased fibrinogen (6.48 g/L), d-dimer (2.21 μg/mL), and a positive SARS-CoV-2 PCR. Non-contrast head CT revealed an acute intraparenchymal hemorrhage of the parasagittal parietal region and subdural hemorrhage of the right posterior fossa (Fig. 1A, B). CVST was suspected, and a contrast head magnetic resonance venography (MRV), with phase-contrast technique, was conducted, which revealed thrombosis of the superior sagittal sinus and right transverse, sigmoid, and trolard sinuses (Fig. 1C–F). A diagnosis of CVST associated with COVID-19 and hyperthyroidism was made. The patient had no other known risk factors for CVST. We have also conducted work-up for other possible causes of thrombophilia, which returned as normal, including normal protein S (109.0%), protein C (79.5%), anti-cardiolipin antibody (ACA) IgG (2.00 GPL U/mL), and ACA IgM (1.20 MPL U/mL), thereby excluding protein S and protein C deficiency, as well as antiphospholipid syndrome.

**Fig. 2:**
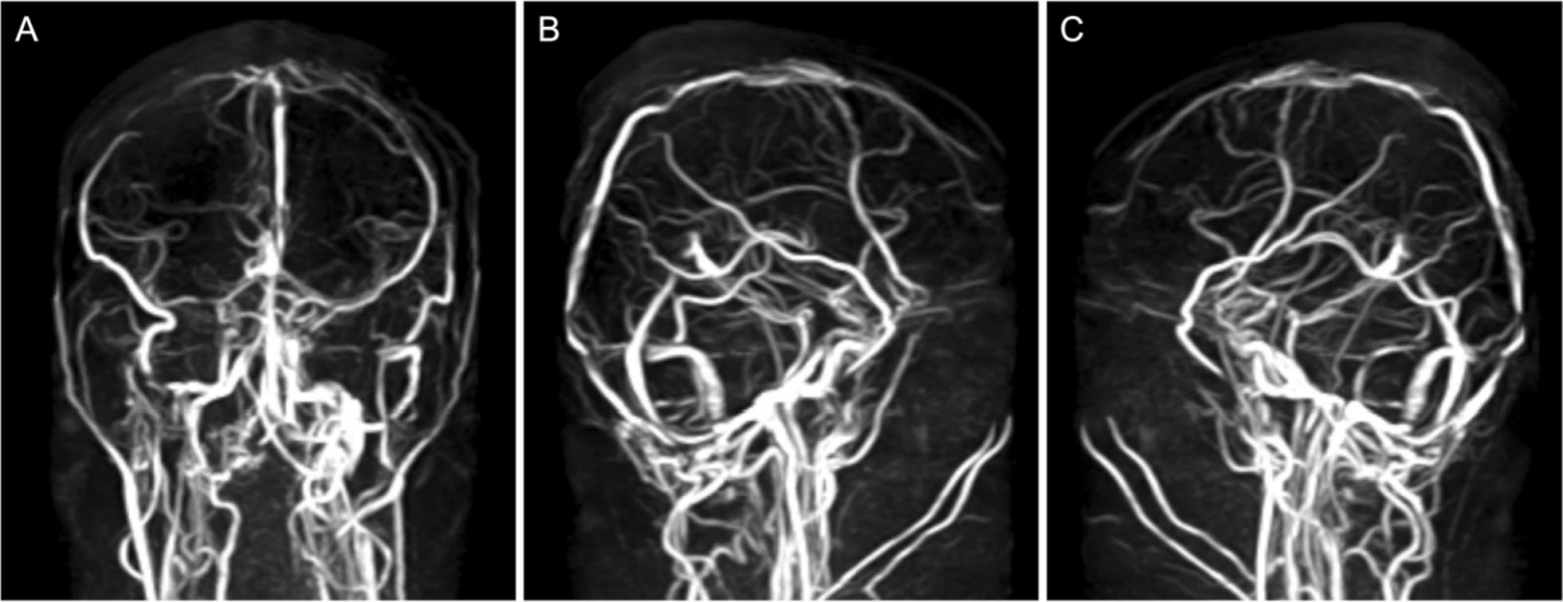
Follow-up head MRV conducted 4 weeks later revealed improvement of thrombosis, with restoration of flow in the superior sagittal sinus (**A**–**C**)

The patient was administered a 3-day course of enoxaparin, after which long-term anticoagulation was initiated with rivaroxaban. The patient was prescribed favipiravir and ivermectin to treat for SARS-CoV-2 infection and continues oral thiamazole for hyperthyroidism. The patient was discharged after 4 days of care. Upon a 2-week follow-up, there was marked symptomatic improvement (VAS 3/10). After 4 weeks, the patient reports complete resolution of symptoms and MRV revealed improvement of thrombosis with restoration of flow (Fig. 2). The patient continues anticoagulation therapy with rivaroxaban.

## Discussion

In COVID-19, CVST may occur in COVID-19 due to virus-associated thrombophilia, virus-mediated hypercoagulable state, or an overzealous host immune response, as suggested by elevated hypercoagulability and inflammatory markers [6]. Extreme elevations of d-dimer levels have been observed in many COVID-19 patients [7] and marked elevations in d-dimer levels have been reported in COVID-19 patients with ischemic stroke [8]. Two studies by Varga and Ackermann et al. uncovered evidence of endotheliitis with associated intracellular viral particles in tissues of the lungs, heart, kidneys, liver, and small intestine, suggesting that SARS-CoV-2 can induce endothelial dysfunction via direct endothelial invasion. It is postulated that SARS-CoV-2 could infect endothelial cells via the angiotensin-converting enzyme 2 (ACE2) receptors present on the cell surface [9, 10]. Another possible route is through an indirect mechanism. In severe COVID-19 disease, an intense systemic inflammatory response towards the virus could result in a hyperinflammatory state otherwise known as a cytokine storm. In such a hyperinflammatory state, the release of proinflammatory cytokines such as IL-6, IL-1, TNF-α, and interferon leads to deleterious effects, including hypercoagulability, endothelial dysfunction, diffuse alveolar damage, multiorgan failure, and death [11, 12].

Incidence of CVST in COVID-19 is exceedingly rare, estimated at 4.5 per 100,000 cases (0.0045%) [5]. Interestingly, the demographic characteristics of patients with COVID-19-associated CVST differ from non-COVID-19-associated CVST patients, as they tend to be predominantly male (56–70%) and older (42–43 years), compared to non-COVID-19 cases, which predominantly affects women in the third decade of life [13–15]. Furthermore, most of the COVID-19 patients affected were relatively young with few comorbidities, and a significant proportion of CVST patients displayed only mild to moderate severity of disease, indicating that COVID-19-associated hypercoagulability may be present even in mild infection [5].

In this patient, however, SARS-CoV-2 infection is not the sole precipitator of CVST. Hyperthyroidism can induce a hypercoagulable and hypofibrinolytic state and has been implicated as a rare predisposing factor for CVST. Retrospective studies of CVST patients in whom thyroid parameters were available found hyperthyroidism was present in 1.9–7.1% of patients [3, 4]. Previous meta-analyses found that high thyroid hormone levels, both in subclinical and overt hyperthyroidism, were associated with elevations in factors VIII, IX, X, Von Willebrand factor, and fibrinogen. In addition, a hypofibrinolytic state in hyperthyroidism may be caused by reduction of plasmin and plasmin activator, and elevation of plasminogen activator inhibitor-1, 2-antiplasmin, and thrombin activatable fibrinolysis inhibitor [16, 17]. While rare, there have been several reports of thyroid dysfunction in CVST. A previous study of 107 CVST patients in whom thyroid measurements were available found 17.8% had thyroid dysfunction [4]. Another systematic review found 34 cases of venous thrombosis in overt hyperthyroidism, of which CVST composes 73.5% of cases [18].

As both hyperthyroidism and COVID-19 are capable of inducing inflammatory and hypercoagulable states to certain degrees, we postulate that COVID-19, even in mild disease, could trigger CVST by superimposing endotheliitis and inflammation on the hypercoagulable and hypofibrinolytic state induced by hyperthyroidism. Finally, we recommend that a high index of suspicion must be maintained in evaluating a COVID-19 patient with headache. Headaches account for 37.7% of neurological symptoms in COVID-19 [19], and are present in 25.2% of all COVID-19 cases [20]. While previous studies found that most headaches in COVID-19 are benign [21, 22] they may also herald a serious underlying etiology, including CVST.

## Conclusions

To the extent of the authors’ knowledge, there have been no previous reports regarding the occurrence of CVST in concomitant COVID-19 and autoimmune hyperthyroidism. Clinicians should be aware that even mild COVID-19 could be sufficient to precipitate CVST, especially in the presence of other risk factors, including autoimmune hyperthyroidism. We recommend that further studies (such as case–control studies) be conducted to evaluate the effects of mild COVID-19 on other prothrombic-inducing conditions, including autoimmune hyperthyroidism. Furthermore, a high index of suspicion toward a secondary cause must be maintained for headaches in COVID-19, as it may indicate a serious etiology, including CVST.

## Data Availability

The patient data described in this case report are not publicly available due to patient privacy concerns but may be available from the corresponding author (rocksy.situmeang@lecturer.uph.edu) upon reasonable request.

## Abbreviations

ACA: Anti-cardiolipin antibody
ACE2: Angiotensin-converting enzyme 2
aPTT: Activated partial thromboplastin time
COVID-19: Coronavirus disease 2019
CRP: C-reactive protein
CT: Computed tomography
CVST: Rebral venous sinus thrombosis
ICH: Intracerebral hemorrhage
IL-1: Interleukin 1
IL-6: Interleukin 6
MRV: Magnetic resonance venography
PCR: Polymerase chain reaction
SARS-CoV-2: Severe acute respiratory syndrome coronavirus 2
SDH: Subdural hemorrhage
TNF-α: Tumor Necrosis Factor alpha
TSHs: Thyroid stimulating hormone (sensitive)
VAS: Visual analogue scale
